# Vacuum Technology Considerations For Mass Metrology

**DOI:** 10.6028/jres.116.014

**Published:** 2011-08-01

**Authors:** Patrick J. Abbott, Zeina J. Jabour

**Affiliations:** Mechanical Metrology Division, National Institute of Standards and Technology, Gaithersburg, MD 20899-0001

**Keywords:** kilogram, mass, vacuum

## Abstract

Vacuum weighing of mass artifacts eliminates the necessity of air buoyancy correction and its contribution to the measurement uncertainty. Vacuum weighing is also an important process in the experiments currently underway for the redefinition of the SI mass unit, the kilogram. Creating the optimum vacuum environment for mass metrology requires careful design and selection of construction materials, plumbing components, pumping, and pressure gauging technologies. We review the vacuum technology^1^ required for mass metrology and suggest procedures and hardware for successful and reproducible operation.

## 1. Introduction

Vacuum mass metrology enables the most precise experimental measurement of the “true mass” [1] of a standard mass artifact by eliminating the need for the air buoyancy force correction and its associated uncertainty. Vacuum mass metrology has received great interest in connection with the internationally coordinated efforts to redefine the SI unit of mass, the kilo-gram, in terms of an unvarying physical constant. In this regard, two experiments performed in vacuum have emerged as leading candidates: the watt balance, which measures the Planck constant [2, 3] and the Avogadro constant project [4]. Both of these experiments will produce a vacuum-referenced definition of the kilogram that will have to be tied to the current International Prototype Kilogram (IPK) [5, 6] in order to maintain continuity and stability for mass metrology that is practiced in air. Several efforts are underway to develop a *mise en pratique*, or practical method of disseminating the vacuum-based kilogram to air [7, 8, 9] all of which require high precision mass balances to operate within an easily reproducible vacuum environment.

The ceaseless demands of the semiconductor industry over the past 40 years have enabled vacuum technology to evolve to the point where high quality materials and components are readily available worldwide at reasonable expense. There are so many off-the-shelf choices of plumbing components, construction materials, pumps, pressure gauges, sealing systems, valves, and feedthroughs that even the most well intentioned designer can make critical mistakes that will compromise the performance of the vacuum system and ultimately affect the quality of the mass measurements made within. It is our purpose to present the basics of vacuum technology required for sound design and construction of a vacuum system suitable for use in mass metrology. We will assume that a high precision, vacuum compatible mass balance is available and that provisions exist for mounting it in the vacuum chamber.

## 2. The Vacuum Environment

### 2.1 Degree of Vacuum

“Vacuum” is a qualitative term used to describe the absence of air pressure within a given volume. It is inversely related to the quantitative term “pressure,” which is a measure of force per unit area. Therefore, we cannot talk about “measuring vacuum,” as it is not a quantitative term, but instead we must measure pressure either directly or indirectly. The SI unit of pressure [10] is the pascal, which is defined as one newton per square meter (1 Pa = 1 N/m^2^). There are many non-SI pressure units, including the torr and millibar (1 Torr = 133.322 Pa, 1 mbar = 100 Pa) in common use, but this paper will use the pascal and other SI units exclusively. The terms “high vacuum” and “low pressure” are used in common parlance with nebulous meanings. Table I relates common understanding of degree of vacuum to quantitative pressure values [11].

In order to calculate the degree of vacuum required for mass metrology, we consider the fact that air or any other gas will exert a buoyant force on a mass artifact that is equal to the weight of the volume of gas that is displaced by the artifact. Assuming the density of stainless steel is 8.0 g/cm^3^, the volume of a 1 kg stainless steel mass artifact is approximately 125 cm^3^; in atmospheric pressure air, the weight of this displaced volume is about 0.15 g. In order to reduce the buoyant force to a negligible level, its magnitude should be much smaller than the uncertainty of the artifact’s mass measurement. Assuming the expanded uncertainty [12] on a 1 kg artifact is approximately 0.050 mg, and that a buoyancy contribution of one percent or less of this value is negligible, the ideal gas equation can be used to calculate the desired pressure within the vacuum vessel, which turns out to be about 0.1 Pa (or less). This is the upper limit of the high vacuum (HV) regime, as shown in Table I above.

Another important consideration for vacuum mass measurement is the number density and species of the gas molecules inside the weighing chamber. Assuming a temperature of 22 °C, a pressure of 10^−1^ Pa, and a sticking probability of unity, a monolayer of air (essentially oxygen and nitrogen) will form on the chamber surface in about 2.5 ms [11]. Air molecules are very weakly physisorbed (desorption energy less than 40 MJ/kg mole) onto a metal surface such as found on the inside of a vacuum chamber and all of the air molecules are easily pumped away. At 10^−1^ Pa, there is a density of about 10^13^ molecules/cm^3^, which is composed mostly of water vapor for an unbaked chamber that has recently been exposed to the atmosphere (atmospheric air at 22 °C and 50 % relative humidity has a water vapor partial pressure of about 840 Pa.). Water molecules are chemisorbed (desorption energy of 96 MJ/kg mole) to a metal surface and have residence times on the order of 30 hours. At 10^−1^ Pa a monolayer of water adsorbs to a metal surface in less than 2 ms. Up to 100 monolayers of water may build up on the surface when exposed to atmospheric pressure air [13] but the binding energy between water monolayers is much less than between water and the metal surface, meaning that almost all but the last monolayer will readily pump away. One hundred monolayers of water molecules on a 1 kg weight can add more than 0.050 mg to the total mass of the artifact; in addition, multiple monolayers of water on the other metallic parts of the balance’s weighing mechanism will add mass in proportion to their area as well. For this reason, it is necessary to allow the vacuum chamber to pump until all but the last monolayer has been removed, which may take several days at room temperature (22 °C). This can be monitored by making multiple weighings over time until the the artifact’s mass is stable to the desired level. Note that the artifact will appear to lose mass as water desorbs and is pumped away. The most effective way of ridding a metal chamber of adsorbed water is by raising the temperature to over 100 °C for an extended period of time; even a slightly elevated temperature of 70 °C will hasten the desorption process. However, this is not possible or advisable for many mass-in-vacuum measurement systems due to the delicate construction of the balance. Most elastomeric seals will tolerate baking temperatures to at least 80 °C, with some materials such as Viton^®^ A [14] able to operate at temperatures well in excess of 100 °C. The considerations presented above suggest that the vacuum balance and mass artifacts under test be subjected to several days of pumping prior to making mass measurements in order to minimize the amount of adsorbed water on the balance and artifacts’ surfaces.

### 2.2 Vacuum System Design and Construction

Several factors must be considered when choosing the vacuum technology used in a given application. Among these are desired ultimate pressure, required degree of cleanliness, whether or not the system will be baked, frequency of cycling to atmospheric pressure, the need to insert and/or remove things while under vacuum, and of course monetary budget. This final criterion is critical; vacuum design is very much driven by the “level of effort” required to achieve system goals. One can build an inadequate system by spending too little or too much for the wrong technology for a given application.

The equilibrium pressure within a vacuum chamber is a balance between the flow of gas into the chamber and the rate at which it is removed. The time required to evacuate the chamber to a given pressure depends upon the chamber volume, the pumping speed of the pump(s), the conductance of the components (tubes, valves, etc.) between the pump(s) and the vacuum chamber, and the influx of gas through the mechanisms of leaks in the chamber, outgassing of materials, virtual leaks (trapped gas within tiny volumes that slowly pumps away), and permeation of atmospheric gases through seals. Vacuum weighing introduces the additional gas load due to the mass balance, which in most cases is unknown and difficult to estimate. The manufacturer of the mass balance that is intended for use in vacuum should take appropriate steps to eliminate as many sources of gas within the balance as is practicable. These steps may include eliminating the application of paint on the outside surface, using high vacuum compatible insulation on wires, eliminating pockets of trapped gas by venting screws, and minimizing the use of “gassy” materials such as plastics, elastomers, and certain lubricants. Given the ambiguity with which the mass balance gas load is known, it is prudent to conservatively estimate the gas load so that sufficient pumping can be incorporated to permit an equilibrium pressure in the desired range. This paper cannot give an in-depth treatment of all of the vacuum system design issues, but references will be given throughout so that the reader can delve deeper into any particular topic.

#### 2.2.1 Materials for Vacuum Systems

First and foremost, a vacuum chamber must be strong enough to support the differential weight of the earth’s atmosphere, which is about 10 N/cm^2^ (15 lb/in^2^). Modern vacuum chambers are typically made from metal, either stainless steel or an aluminum alloy, and may be 5 mm or more thick depending on the surface area. The ASME has published standards for the wall thickness of cylindrical vessels under external pressure [15]; easy to use charts that provide wall thickness as a function of material and diameter for cylindrical and spherical chambers may be found in the references [16, 17]. Engineers for commercial vacuum component manufacturers can also assist in selecting the right material and thickness for a given chamber. Both aluminum and stainless steel offer high strength, cleanliness, weldability for easy modification, and “building block” component systems that make vacuum system design straightforward. The glass vacuum systems that were common prior to 1970 are exceedingly rare nowadays except for highly specialized applications or demonstrations that use glass bell jars. There are many grades of stainless steel, not all of which are suited for use in vacuum chamber construction [18]. The properties of good weldability, high temperature bakeability, relatively low cost, and low contaminant outgassing make 304 stainless steel a good choice. A variety of weldable flanges and other fittings are available in a wide range of standardized sizes and sealing systems [19], making component selection very versatile and convenient. Aluminum of the 6000 series is also used for vacuum chamber construction but is generally restricted to medium and high vacuum systems that use elastomer sealed flanges; ultrahigh vacuum aluminum and stainless steel bimetal seals are also available at greatly increased cost [20].

In theory, a vacuum chamber can be made in any shape, though easy to fabricate vessels such as right circular cylinders are most common. In applications where minimizing surface area (and hence outgassing) is important, spherically shaped chambers are used. Special surface treatments such as electropolishing provide a high luster finish that further reduces surface area. This is important for ultrahigh vacuum applications, but not critical for high vacuum applications. A variety of coating processes are also available to reduce outgassing [21] or enhance pumping, but again these are geared to UHV applications.

Every vacuum system contains an experiment that must be monitored and interacted with from outside the vacuum environment. Environmental sensors such as temperature and pressure, electrical connectors, and various types of motion feedthroughs are some commonly used items that need some way of attaching to the vacuum chamber. As mentioned earlier, ports and flanges of many shapes, sizes, and functions are commercially available to interface to a vacuum chamber through standard fittings. The designer should consider the type and quantity of feedthroughs and ports needed for temperature, pressure, and humidity measurements *before* the chamber is constructed.

Electrical grounding is a very important safety and performance consideration. A metal chamber should be grounded to a “true” ground (earth) that doesn’t fluctuate with electrical noise from other instruments. Safety grounds found on U.S. 3-pronged outlets are typically inadequate. Direct connection to a metal cold water pipe that is buried in the earth is best. All other instruments in use should have the same ground as the chamber to avoid ground loops.

### 2.2.2 Sealing Options

Mass metrology requires a high vacuum system that can be exposed frequently to atmospheric pressure. The bakeability of the chamber is limited by the ability of the mass balance, artifacts, and seals to withstand heat. In general, it is not advisable to expose high precision mass comparators to elevated temperatures. At least one manufacturer [22] of these instruments specifies the operating temperature range to be between 17 °C and 30 °C (with an upper limit of 27 °C for some models), so effective baking of the chamber to remove adsorbed water is not possible. Given these constraints, elastomer sealed vacuum flanges are sufficient using either aluminum or 304 stainless steel construction.

Elastomer O-rings vary in their chemical composition. Viton^®^ A, Neoprene, Kalrez^®^ [23] and Buna N (also called Nitrile) are common elastomers used for vacuum seals [24]. Of these, Viton^®^ A, a fluoroelastomer, is generally considered to be the “cleanest” in terms of outgassing rate and the most versatile, as it can be used at elevated temperatures of up to150 °C. Viton^®^ A and many other elastomers are hygroscopic, absorbing moisture from the air at atmospheric pressure and then releasing it under vacuum. These elastomers also allow the permeation of atmospheric gasses into the chamber while under vacuum; for these reasons, the water outgassing rate and permeation rate of elastomer seals limits the ultimate pressure of the vacuum chamber and plays a large role in pump specifications. The outgassing rate of elastomer seals can be reduced by prebaking O-rings to 100 °C [25]. Unlike metal sealed systems, water cannot be eradicated from elastomer sealed systems due to the constant influx from permeation of atmospheric water. For this reason, water is the primary component of the elastomer sealed high vacuum environment. This is an important consideration in mass metrology, as water adsorbed onto mass artifacts is the subject of much research [26, 27, 28, 29].

**Fig. 1:**
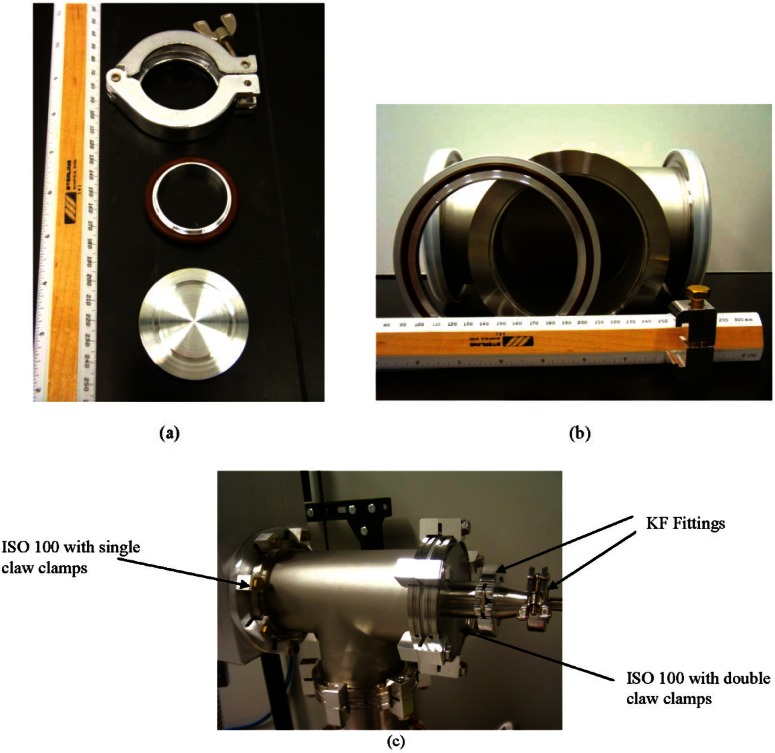
Photos of elastomer sealing systems. (a) KF or NW, showing (top to bottom) clamp, retaining ring and gasket, and blank fitting), (b) ISO 100, showing (left to right) retaining ring and gasket, tee-flange, and double claw clamp, (c) a vacuum tee in the authors’ lab having both KF and ISO 100 flanges.

The ISO (International Organization for Standardization) elastomer sealing flanges [30], such as “Quick Flange” (QF, KF, or NW are common designations [31]), are convenient for use in high vacuum chamber construction and are available through many manufacturers. Photos of each of these sealing systems are shown in Fig. 1. Both ISO and QF systems use an elastomer O-ring held in place by a metal retaining ring; the retaining ring/O-ring assembly is placed between two flat flanges and clamped together via nuts and bolts, claw clamps, or in the case of QF, special clamps that encompass the perimeter of the mating flanges. The clamps should be tightened to the specified torque values for ISO clamps. For QF clamps there is a wing nut or a “T” shaped nut that is used to tighten the clamp; this should never be tightened any more than finger tight. In no situation should tools like pliers or a wrench be used to tighten these fittings. Over-tightening can cause the O-ring to exceed the compression ratio specified for sealing and lead to a faulty seal. Vacuum grease should not be applied to the O-rings of these seals; no sealing issues should arise if all flange surfaces are clean, parallel, and free of burrs, scratches, and other defects. The use of grease may actually inhibit a good seal, as the proper O-ring compression may not be achieved with the specified clamping torque. In general, the use of vacuum grease should be avoided as it can migrate into the vacuum chamber and possibly contaminate weights, comparator parts, or other delicate instrumentation.

### 2.3 Sources of Gas in Vacuum Systems

There are many sources of gas in a real vacuum chamber. The following are the most common sources [32].

- Outgassing from adsorbed layers
- True Leaks (holes or cracks)
- Porosity of metals
- Virtual leaks (pockets of gas trapped by screws, spacers, etc.)
- Permeation from internal surfaces (elastomers, metals, etc.)
- Outgassing of surface contamination (hydrocarbons)
- Pressure gauges (especially hot cathode)
- Backstreaming from the pumping system.

**Fig. 2:**
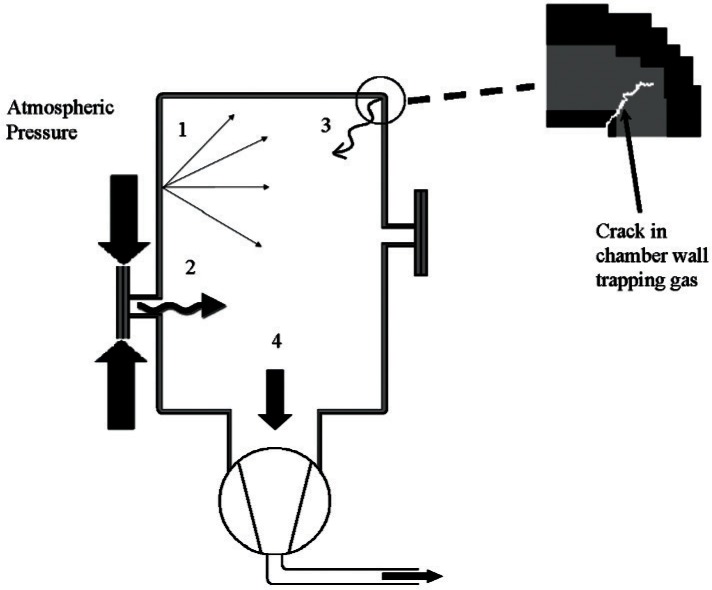
Illustration of the major sources and sinks of gas in a vacuum chamber; arrows represent the flow of gas molecules (1) desorption and outgassing from surfaces; (2) permeation from the atmosphere through seals; (3) “virtual leaks” of trapped gas; (4) evacuation through chamber pump(s).

It is beyond the scope of this paper to fully describe each of these, and the reader is encouraged to consult the references listed at the end. A diagram showing the major sources and sinks of gas in a vacuum chamber is shown in Fig. 2. In an unbaked metal chamber with metal seals and sufficient pumping, it is possible to remove most gas molecules except the chemisorbed water on the chamber walls. This typically results in a base pressure of approximately 10^−6^ Pa. If elastomer seals are used, a steady stream of atmospheric gases will permeate the O-rings and will limit the base pressure to around 10^−5^ Pa, and higher than this depending on the number and size of the O-rings. For an unbaked chamber, water will desorb from the metal surfaces within the chamber at a very slow rate, thus adding to the gas load of the elastomers. In order to minimize the outgassing rate inside the chamber, the size and number of elastomer gaskets should be kept to a minimum, as the outgassing rate is proportional to the total surface area of all the gaskets combined. Don’t use large dia meter ports where smaller ports will do. Generally it is a good idea to have a few spare ports for future considerations, but too many will increase the chamber’s gas load if elastomer seals are involved.

A general expression for the equilibrium pressure within the chamber, *P_eq_* is:

(1) $$P_{eq}=P_{ult}+\frac{Q_{og}}{S_{net}}.$$

In (1), *P_ult_* is the ultimate pressure (lowest possible) for the pump, *Q*o*_g_* is the *total* outgassing rate in Pa m^3^/s, and *S_net_* is the net pumping speed for the system in m^3^/s. This equation tells us that there are two ways to lower the equilibrium pressure in the chamber: by reducing the outgassing rate and/or by increasing the net pumping speed.

Contaminants such as hydrocarbons from pump oil, dirt, and fingerprints will all outgas and limit the chamber’s ultimate pressure. To minimize contamination, the inside surface of the vacuum chamber should be cleaned with an alkali detergent and thoroughly rinsed with deionized water. Any parts to be inserted into the chamber should also be cleaned with detergent and deionized water or reagent grade organic solvents such as acetone and ethyl alcohol if grease or oil is present. Note that parts should be completely dried after cleaning and prior to installing within the vacuum chamber. Any solvent trapped in the vacuum chamber during pumping will present an enormous gas load to the pump and will greatly lengthen the time needed to reach an acceptable pressure. For this reason, elastomers should never be cleaned with solvents, as they are absorbed by the elastomer and will outgas in the vacuum chamber during pumping. In order to keep a vacuum chamber clean, precautionary measures need to be taken. The laboratory environment should be kept clean of dust and dirt that could migrate into the vacuum chamber when a port is opened. The length of time a chamber is open to the atmosphere should be minimized to reduce the quantity of gas that is adsorbed and absorbed by chamber components. While working on the inside of the chamber, or on any parts that will be put inside the chamber, clean, lint-free gloves should be worn to prevent transfer of dirt and oil from fingers and hands. Latex or nitrile gloves such as those worn in the health profession work well, and it is best to remember that gloves are only as clean as the last item they touched. For prolonged work inside a vacuum chamber, change gloves often. Powdered gloves should never be worn. If vacuum ports must be open for long periods of time, then it is prudent to cover them with a plastic protective cap or with *clean* aluminum foil. For high vacuum applications such as mass measurements, commercial aluminum foil is sufficiently clean; however ultra-high vacuum applications require a higher degree of cleanliness, and traces of oils used for lubricating the foil during the rolling process may contaminate the vacuum system and limit the achievable base pressure. Venting from vacuum to atmospheric pressure should be done with a clean, dry, non-reactive gas. Filtered air and nitrogen are good choices.

### 2.4 Pumping Considerations

#### 2.4.1 Types of Vacuum Pumps

The purpose of a pumping system is to remove gas from the vacuum chamber. The rate of gas removal should be much greater than the flow rate of gas into the chamber in order to achieve the desired pressure in a reasonable amount of time. There is no single pump that can evacuate a chamber from atmospheric pressure down to high vacuum; instead, a combination of differ ent pumping technologies must be used. Initial evacuation from atmospheric pressure down to about 1 Pa, sometimes referred to as “roughing,” can be obtained using a variety of primary or “mechanical pumps” including oil sealed rotary vane pumps, piston pumps, diaphragm pumps, or scroll pumps. For details on each of these designs, the reader is referred to recent texts on vacuum technology [33, 34, 35]. Other than the oil sealed rotary vane pump, the other pumps mentioned above are “dry pumps,” meaning that they are oil free; this is a big advantage in that these pumps do not require the use of a trap to prevent condensable oil vapors from backstreaming from the pump to the vacuum chamber. Contamination from pump oil back-streaming can be catastrophic for the vacuum chamber and any equipment inside the chamber, such as mass balances and weights.

In choosing a primary pump, the variables are pumping speed, cleanliness, and cost. For mass metrology in vacuum, the use of any type of primary pump containing sealing oil is *strongly* discouraged due to the possibility of oil contamination from backstreaming. Scroll pumps [36] have the highest available pumping speed of any dry roughing pump, are compact, and are competitively priced with oil sealed rotary vane pumps. Maintenance of the tip seals must be performed yearly for heavily used scroll pumps, but replacement tip seal maintenance kits are available from vendors and are easily installed. An isolation valve between the scroll pump and the vacuum chamber (or high vacuum pump) must be part of the design of the pumping system in order to prevent possible particulate migration from the scroll pump to the vacuum chamber in the event of loss of electrical power [37].

**Fig. 3:**
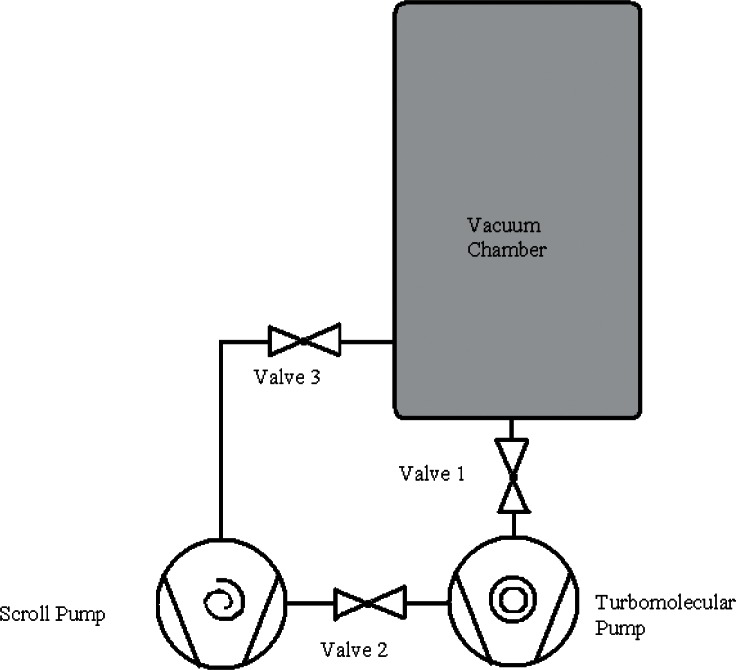
A vacuum chamber using a scroll pump as both primary pump and backing pump for a turbomolecular pump.

A scroll pump has an ultimate pressure of about 5 Pa; to achieve lower pressures, it is necessary to use a high vacuum pump in combination with the scroll pump. Modern turbomolecular pumps having a molecular drag stage [38], i.e., turbodrag pumps, are efficient, clean, reliable, quiet, and can be mounted in any orientation. Turbodrag pumps come in many sizes and are available in pumping speeds from a few liters per second to many thousands of liters per second. Depending on the size of the chamber and the desired pressure, a typical turbodrag pump for a mass-in-vacuum application will have a nominal pumping speed of 100 L/s to 500 L/s. Turbodrag pumps compress the gas at their inlets and require a backing stage to remove the compressed gas from their exhaust ports. The design shown in Fig. 3 uses the same primary chamber pump as the turbo-molecular pump’s backing stage.

There are other types of high vacuum pumps, but they are impractical for vacuum mass metrology. Diffusion pumps use jets of oil vapor to transfer momentum to gas molecules in order to pump them away. Cold traps are necessary to prevent oil contamination of the vacuum chamber. They also require high temperatures to vaporize the oil, which may cause thermal gradients in the vacuum system. Unlike the turbomolecular and diffusion pumps which physically move molecules from the vacuum chamber to a rough pump, ion pumps use an ion burial gettering process to remove gas molecules from the chamber. The molecules are buried in the pumping elements which have a finite ability to pump gas. Therefore, ion pumps are practical only for very small gas loads, corresponding to chamber pressures typically less than 10^−5^ Pa. In addition, potentially lethal voltages of several thousand volts and very high magnetic fields are required to operate ion pumps.

#### 2.4.2 Conductance

The efficacy of a given pump in evacuating a metal chamber depends not only on the design of the pump, but also on the “plumbing” used to connect the pump to the chamber. The diameters of the connecting chamber port and connecting pipes, the number and severity of bends in the connecting hardware (such as with elbows), and the total length of pipe between the chamber and the pump affect the overall pumping speed through their *conductance* [39, 40, 41]. Conductance is a measure of the speed with which a unit volume of gas is moved through a given passageway; in Ref. [24], Dushman refers to conductance as the rate of flow per unit difference of pressure. The units of conductance are the same as those of pumping speed, that is, volume per unit time (e.g., or m^3^/s or L/s). The larger the diameter (cross sectional area) of the connecting pipe, the higher the conductance, and the faster the gas molecules will be pumped away. For good conductance, the pipe connecting the high vacuum pump with the chamber should have a wide diameter, no bends, and be as short as possible. In general, it is good practice to use connecting hardware having about the same diameter as the high vacuum pump’s inlet port. Practically, this is limited to diameters of around 100 mm (about 4 inches) for ISO-100 fittings. Small diameter connecting hardware in effect “chokes” the high vacuum pump, and the effect on pumping speed is analogous to draining a swimming pool with a drinking straw.

Conductance can be maximized by fastening the inlet port of the high vacuum pump directly to an equal diameter port on the vacuum chamber. However, this is a risky proposition with mass metrology in that vibrations from the pump may couple into the sensitive mass comparator and introduce a source of instability. While it is good vacuum design practice to keep the pumps as close as possible to the vacuum chamber, vibration isolation measures may need to be considered, such as adding a bellows between the pump and chamber or simply locating the pump a moderate distance away from the chamber. Davidson [9] has eliminated the vibration problem by using a turbo-molecular pump having magnetically levitated blades. Using a wide diameter pipe (100 mm is usually sufficient) to locate the pump a few meters from the chamber will reduce the effect of vibrations and heat that are generated by both the primary and high vacuum pumps. The connecting pipe should have as few bends as possible, as each bend reduces the total conductance. The effective conductance between pump and chamber may be calculated for a given vacuum system, but the analytical expressions change as a function of gas flow regime. The formulae for making these calculations may be found in any of the vacuum technology texts appearing in the reference section. In most cases, the above sources of gas, along with component conductances and pumping speeds can be calculated, measured, or estimated with sufficient accuracy in order to create an effective design.

## 3. Measuring Pressure

### 3.1 Types of Pressure Gauges

Pressure is force per unit area, and is commonly measured over 16 decades from about 10^9^ Pa to 10^−7^ Pa. To make accurate measurements over such a wide range requires many different technologies. Pressure gauges may be direct or indirect reading. In direct reading gauges, a non-gas dependent change in some physical property of the gauge occurs in response to the application of pressure. An example of this type is the capacitance diaphragm gauge [42], in which a thin metal diaphragm stretched between two electrodes deflects away from its equilibrium position with the application of pressure; the deflection changes the capacitance between the diaphragm and the electrodes, and this change is converted to a pressure indication by conditioning electronics. Indirect reading gauges respond to some other quantity that is proportional to pressure and frequently dependent on gas species. An example of this is the thermocouple gauge which measures heat flow as a function of pressure [43]. The type of pressure gauge used in a particular application depends on the pressure range of interest, the desired accuracy of the measurement, compatibility with other apparatus, and of course monetary cost. Table II lists some of the more common gauges that are useful for mass in vacuum metrology along with some of their characteristics.

### 3.2 Pressure Gauge Calibration

Table II presents accuracy estimates for uncalibrated gauges, and these estimates can vary widely according to gauge type [44]. Thermal conductivity gauges and ionization gauges may be inaccurate by as much as a factor of two. For proper use, any gauge should be calibrated prior to making measurements. A calibration will provide the user with a pressure-dependent correction factor to apply to the gauge’s indicated pressure value, as well as an uncertainty estimate on the corrected pressure values *at the time of calibration*. The uncertainty estimate is critical, and the user should not expect better results than the calibration uncertainty dictates. The user must always operate the pressure gauge in a manner consistent with its calibration; failure to do so will void the calibration. Calibration conditions to observe include using the exact same hardware as was used for the calibration (e.g., one can’t mix and match gauge controllers with gauge heads in general), proper operating parameters, the same gas as the gauge was calibrated with, mounting the gauge in the proper orientation, and observing the safe pressure range of the gauge. Even with proper calibration, the response of many pressure gauges tends to drift away from calibration with time and use. Several factors influence this drift including exposure to atmospheric pressure, accidental over pressurization, rough handling, or inherent limitations of the gauge’s technology. For instance, inexpensive thermocouple gauges may not be stable to within 20 % even over the course of the calibration! It is essential to choose a gauge and calibration service that is appropriate for the application. Many vendors offer calibrations that are traceable [45] to the National Institute of Standards and Technology (NIST) or other National Metrology Institutes. Only history and experience can determine how frequently your pressure gauges need to be calibrated. It cannot be overstated that when choosing a calibration service, it is prudent to be mindful of the required uncertainty for the pressure measurement application. For mass-in-vacuum metrology, the calculation above determined that the upper limit of pressure is 0.1 Pa. To measure the difference between 0.1 Pa and 0.09 Pa, it’s necessary to have better than 10 % uncertainty. Typically, lower calibration uncertainty translates into higher gauge cost. In this case, paying for a gauge that can hold a 0.1 % calibration uncertainty is not necessary when 1 % is more than adequate. The calibration of the gauge becomes less critical at lower pressures, but the operation of certain balances might be affected.

### 3.3 Pressure Gauge Recommendations

The pressure measurement requirements for mass-in-vacuum call for a clean, stable gauge to monitor pressure during evacuation and to have less than 10 % uncertainty at the pressure of interest where the mass metrology will be performed. In addition to uncertainty constraints, the gauge should not generate any contaminants or an excessive amount of heat. A convection enhanced Pirani gauge is adequate to a pressure of 0.1 Pa, but may be unreliable at lower pressures. In the same way, thermocouple gauges suffer from large uncertainties at their lowest range, around 0.1 Pa. Ionization gauges, both hot cathode and cold cathode, are at their high pressure limits at 0.1 Pa and are greatly influenced by space charge effects. Furthermore, there is evidence that contamination produced by the ionization process in a cold cathode gauge can influence mass measurements [46].

**Fig. 4:**
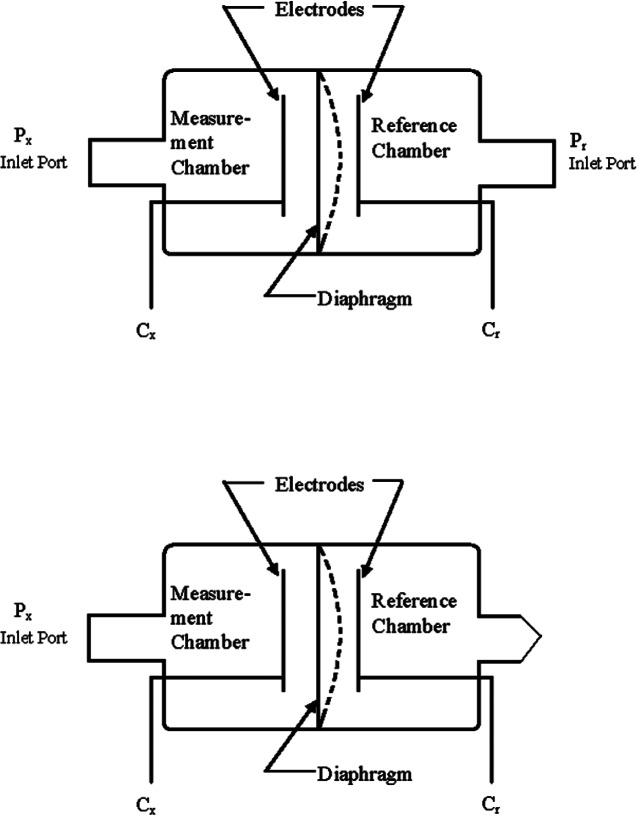
Diagrams of differential (top) and absolute (bottom) capacitance diaphragm gauges. P_x_ and P_r_ are the unknown and reference pressures respectively. C_x_ and C_r_ are the capacitor plates on the unknown and reference sides (after Hyland and Shaffer, Ref. [40]).

**Fig. 5:**
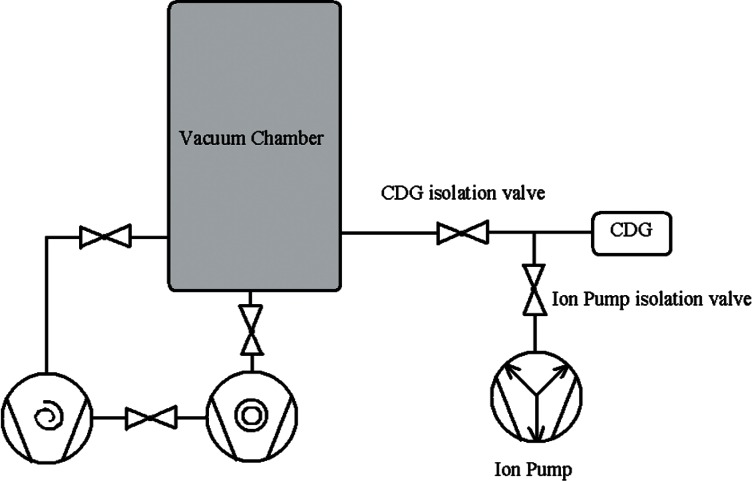
Auxiliary pumping system for zeroing a capacitance diaphragm gauge (CDG) prior to use. The ion pump need only have a pumping speed of 1 or 2 L/s. The CDG is first pumped down to 0.1 Pa or less by the vacuum chamber pump (CDG isolation valve open); the isolation valve is closed and the ion pump is opened to provide a hard zero sufficient for zeroing the CDG.

Capacitance manometers, or capacitance diaphragm gauges (CDG) can cover the pressure range between 10^5^ to 10^−3^ Pa, respond directly to pressure, and when calibrated have an uncertainty of 0.5 % or less [47]. Multiple gauge heads are needed to cover the entire range from atmospheric pressure down to 0.1 Pa or less. This can be done using a minimum of two gauge heads having full scale ranges of 133 kPa and 133 Pa. For pressures from atmospheric to 10 Pa, a convection enhanced Pirani gauge will perform sufficiently and may save money over the use of a 133 kPa CDG. Regarding the use of CDG’s, both differential and absolute models are available, but a differential CDG requires a separate pump to provide a low reference pressure in the high vacuum regime. Absolute CDG’s have a gettering material incorporated into the reference pressure side of the diaphragm that maintains a high vacuum reference pressure. Figure 4 shows diagrams for both absolute and differential models. One drawback of both absolute and differential CDG’s is that they must be zeroed prior to use by applying high vacuum to their measurement ports. If the vacuum chamber is not capable of doing this, then a small auxiliary ion pump can be used to provide this “hard zero” when necessary. A scheme for doing this with a small ion pump is shown in Fig. 5.

In summary, the types of pressure gauges to be used are a trade-off between accuracy, cleanliness, and cost. We recommend that a less accurate (and less expensive) device such as a convection enhanced Pirani gauge or a piezoresistive gauge be used to measure pressure from 100 kPa down to 10 Pa, in conjunction with a more accurate absolute capacitance diaphragm gauge of 133 Pa full scale range to measure pressure down to 10^−2^ Pa and possibly lower. A differential CDG may be used if a reference “zero” pressure is readily available. For the Pirani gauge, it is important to follow the manufacturer’s instructions for initial setup, which is usually a check of the gauge readings at atmospheric pressure and at “zero pressure” (less than 10^−2^ Pa). Convection enhanced Pirani gauges need to be mounted so that the axis of the gauge is horizontal. It is also important to use the Pirani gauge to measure pressures only for the gas with which the gauge was calibrated (air or nitrogen usually) as catastrophic mistakes (falsely low or high pressure readings) can occur when using a gas having a different thermal conductivity than the calibration gas. Capacitance diaphragm gauges need to be properly zeroed prior to use. Their response is independent of the gas used; however, they can be damaged if they are over pressurized; follow the manufacturer’s instructions regarding this issue.

## 4. Conclusions

We have reviewed vacuum technology design considerations that are germane to mass measurement in vacuum and recommend the following: 1) Based on the need to minimize the air buoyancy correction of the mass measurements a maximum chamber pressure of 0.1 Pa should be used for mass metrology. Other factors, such as the amount of water adsorbed onto mass artifacts may dictate that a working pressure lower than 0.1 Pa be used. 2) Scroll pumps are a good choice for initial rough pumping of a vacuum chamber as well as backing a high vacuum pump. They are oil-free, competitively priced and comparable in performance to oil-sealed rotary vane pumps. 3) Turbomolecular drag pumps are the best option for producing high vacuum for mass metrology. They are clean, come in a variety of pumping speeds, and enable the use of oil-free backing pumps. 4) A convection enhanced Pirani gauge is economical and sufficiently accurate for measuring pressure from atmospheric pressure to 10 Pa during initial chamber evacuation. For measuring pressure below 10 Pa, a calibrated capacitance diaphragm gauge offers the necessary accuracy, has good stability, and is contaminant-free. A 133 Pa full scale calibrated CDG can accurately measure pressures down to 10^−3^ Pa with an expanded uncertainty of less than 1 %.

## Figures and Tables

**Table I:** Pressure ranges corresponding to degree of vacuum

Degree of Vacuum (abbreviation)	Pressure Range (Pa)
Low	10^5^ > P > 3.3 × 10^3^
Medium	3.3 × 10^3^ ≥ P > 10^−1^
High (HV)	10^−1^ ≥ P > 10^−4^
Very high	10^−4^ ≥ P > 10^−7^
Ultrahigh (UHV)	10^−7^ ≥ P > 10^−10^
Extreme high vacuum (XHV)	10^−10^ > P

**Table II:** Pressure gauges suitable for mass-in-vacuum metrology

Type of Gauge	Pressure Range	Direct/Indirect	Gas species dependent?	Principle	Accuracy (uncalibrated)	Cost
Thermo-couple	1 kPa – 0.01 Pa	Indirect	yes	Heat transfer	± 20 %	low
Convection Enhanced Pirani	100 kPa – 0.1 Pa	Indirect	yes	Heat transfer	± 20 %	low
Capacitiance Diaphragm Gauge	100 kPa – 10^−3^ Pa	Direct	no	Pressure dependent capacitance change	< ± 1 %	high
Piezoresistive	100 kPa – 10 Pa	Direct	no	Pressure dependent change in resistance	< ± 1 %	medium
Resonant Silicon Gauge	100 kPa – 100 Pa	Direct	no	Pressure dependent change in resonator frequency	± 0.01 %	very high
Hot Cathode (Bayard-Alpert)	10^−1^ Pa – 10^−7^ Pa	Indirect	yes	Ionization of gas molecules	± 25 %	medium
Cold Cathode	1 Pa – 10^−7^ Pa	Indirect	yes	Ionization of gas molecules	± 25 %	medium
“Wide Range”	100 kPa – 10 Pa	Indirect	yes, over at least part of the full range	Combination of thermal conductivity gauge or piezo resistive gauge with hot or cold cathode gauge	< 10 % above ^1^ Pa; ± 20 % below 1 Pa	medium
